# Personalised 3D Printed Medicines: Optimising Material Properties for Successful Passive Diffusion Loading of Filaments for Fused Deposition Modelling of Solid Dosage Forms

**DOI:** 10.3390/pharmaceutics12040345

**Published:** 2020-04-11

**Authors:** Jose R. Cerda, Talaya Arifi, Sejad Ayyoubi, Peter Knief, Maria Paloma Ballesteros, William Keeble, Eugen Barbu, Anne Marie Healy, Aikaterini Lalatsa, Dolores R. Serrano

**Affiliations:** 1 Departament of Pharmaceutics and Food Technology and Instituto Universitario de Farmacia Industrial (IUFI), School of Pharmacy, University Complutense, Avenida Complutense, 28040 Madrid, Spain; jcerda@ucm.es (J.R.C.); sejadayy@gmail.com (S.A.); pballesp@ucm.es (M.P.B.); 2 Biomaterials, Bio-Engineering and Nanomedicine (BioN) Lab, Institute of Biomedical and Biomolecular Sciences, School of Pharmacy and Biomedical Sciences, University of Portsmouth, White Swan Road, Portsmouth PO1 2 DT, UK; talaya.arifi@myport.ac.uk (T.A.); eugen.barbu@port.ac.uk (E.B.); katerina.lalatsa@port.ac.uk (A.L.); 3 UCD Centre for Precision Surgery, Catherine McAuley Education and Research Centre, Dublin 7, Ireland; peter@knief.de; 4 Faculty of Technology, University of Portsmouth, White Swan Road, Portsmouth PO1 2 DT, UK; william.keeble@port.ac.uk; 5 SSPC The SFI Research Centre for Pharmaceuticals, School of Pharmacy and Pharmaceutical Sciences, Trinity College Dublin, Dublin 2, Ireland; healyam@tcd.ie

**Keywords:** 3D printing, fused deposition modelling (FDM), Hansen Solubility Parameters, passive diffusion, filaments, PVA, PLA, nifedipine

## Abstract

Although not readily accessible yet to many community and hospital pharmacists, fuse deposition modelling (FDM) is a 3D printing technique that can be used to create a 3D pharmaceutical dosage form by employing drug loaded filaments extruded via a nozzle, melted and deposited layer by layer. FDM requires printable filaments, which are commonly manufactured by hot melt extrusion, and identifying a suitable extrudable drug-excipient mixture can sometimes be challenging. We propose here the use of passive diffusion as an accessible loading method for filaments that can be printed using FDM technology to allow for the fabrication of oral personalised medicines in clinical settings. Utilising Hansen Solubility Parameters (HSP) and the concept of HSP distances (Ra) between drug, solvent, and filament, we have developed a facile pre-screening tool for the selection of the optimal combination that can provide a high drug loading (a high solvent-drug Ra, >10, and an intermediate solvent–filament Ra value, ~10). We have identified that other parameters such as surface roughness and stiffness also play a key role in enhancing passive diffusion of the drug into the filaments. A predictive model for drug loading was developed based on Support Vector Machine (SVM) regression and indicated a strong correlation between both Ra and filament stiffness and the diffusion capacity of a model BCS Class II drug, nifedipine (NFD), into the filaments. A drug loading, close to 3% w/w, was achieved. 3D printed tablets prepared using a PVA-derived filament (Hydrosupport, 3D Fuel) showed promising characteristics in terms of dissolution (with a sustained release over 24 h) and predicted chemical stability (>3 years at 25 °C/60% relative humidity), similar to commercially available NFD oral dosage forms. We believe FDM coupled with passive diffusion could be implemented easily in clinical settings for the manufacture of tailored personalised medicines, which can be stored over long periods of time (similar to industrially manufactured solid dosage forms).

## 1. Introduction

Modern 3D printing (3DP) techniques have opened a wide range of possibilities for the manufacture of solid dosage forms, in particular where there are clear benefits derived from adjusting the drug dose to the needs of each patient. Dose personalisation is known to maximise the therapeutic efficacy of drugs [1] and to reduce the incidence of undesirable effects, which are critical for drugs with a low therapeutic index (such as anticoagulants e.g., warfarin), cytostatic drugs, or very potent actives [2]. To complement these advantages, 3DP allows for easy personalisation of medicines in terms of composition, colour, and dosage form size [3].

3DP techniques that can be used for oral dosage forms include those based on inkjet printing, nozzle deposition, and laser writing systems. Inkjet printing-based systems require a post-processing heating step to eliminate the solvents used during the process, and residuals can be of major concern [4]; in addition, the resulting printed structures are generally very fragile and have irregular shapes due to the high porosity of the materials [5]. Laser-based writing (stereolithography) systems use UV-induced radical photopolymerisation in the presence of a photoinitiator, which is commonly not GRAS (Generally Regarded As Safe), therefore compromising the safety profile of the solid dosage form [6]. A third category of 3DP includes nozzle-based deposition systems such as fuse deposition modelling (FDM) and pressure assisted syringes (PAM). The latter is widely used for solid dosage forms as it uses biocompatible excipients and can incorporate high amounts of drug into the printed tablets; PAM uses a syringe extruder that deposits the semisolid or viscous material layer by layer, following the designed 3D geometry. This technique allows printing at room temperature, which can be useful for thermolabile drugs, however it frequently requires the use of solvents in order to ensure proper viscoelastic characteristics of the extruded material and this, in turn, necessitates a post-processing step to ensure complete removal of the solvents prior to patient administration. In contrast, FDM is a 3DP technique based on the use of drug pre-loaded filaments that are melted and extruded via a nozzle, being deposited layer by layer.

FDM can generate solid dosage forms of appropriate mechanical strength and dosing accuracy; however, currently, there is only a limited selection of thermoplastic materials approved for human use. In addition, the preparation of personalised drug-loaded filaments requires a hot melt extruder and the application of high temperatures for relatively long periods of time (compared to the FDM 3DP process itself) can result in drug degradation and modification of the polymer characteristics. Finding a suitable drug-excipient mixture can be challenging, and while clinical studies have been performed using 3DP in hospitals [7,8], there is scope to widen the applicability and accessibility of 3DP as a practical and affordable technology for manufacturing personalised solid dosage forms that meet patients’ needs. Understanding the material properties of commercial filaments is, however, key in ensuring an effective drug loading method that ultimately allows for optimal FDM printing of oral dosage forms. Passive diffusion overcomes some of the issues highlighted above that are associated with the HME approach to preparing drug-loaded filaments, however one of the main reasons why there has been a move away from this approach in recent years, and towards HME as a more routine method for loading filaments [9,10], is because lower drug loadings have typically been achieved compared to those obtained by HME. There is also limited accessible information available as to how drug loading may be optimised using a passive diffusion approach. Because of the limited loading that can be typically achieved (in most cases <1–2% drug), passive diffusion is currently applicable only to extremely potent drugs [11,12,13]. However, we believe the loading process can be significantly improved by optimally combining a biocompatible and extrudable polymeric material with a suitable non-toxic solvent, enabling the fabrication of a range of dosage forms and widening the range of applicable drugs.

This work is underpinned by the hypothesis that passive diffusion is still a suitable technique to control the drug loading in printable commercial filaments, resulting in an FDM translational process which applicable and may be easily implemented in hospitals and community pharmacies. We propose a predictive model that correlates the properties of solvents and filament polymeric materials (such as Hansen solubility parameters, roughness, and stiffness) with the steady state flux of drug into the filament on loading. Nifedipine (NFD) was selected as a poorly water-soluble antihypertensive model drug that is widely prescribed, and for which several doses and dosage forms with different release profiles are commercially available. Here, we report the results of our investigations aimed to understand how NFD can be successfully loaded in commercially available 3DP filaments based on biocompatible polymers (such as polyvinyl alcohol PVA, PVA derivatives, and poly-lactic acid PLA). A Hansen Solubility Parameters (HSP) approach combined with Multivariate Analysis (MVA) were applied to understand how material properties affect drug passive diffusion into polymer filaments. 3DP tablet characterisation data are presented together with results of dissolution and stability studies, carried out to demonstrate the feasibility of passive diffusion coupled with FDM in manufacturing NFD tablets with similar characteristics to commercially marketed medicines.

## 2. Materials and Methods

### 2.1. Materials

Nifedipine (NFD) was purchased from Industria Chimica Italiana (>95%, Bergamo, Italia). Four different commercially available filaments of 1.75 mm in diameter were utilised: (i) Hydrosupport (HS), containing >96% PVA and polyethylene glycol, was purchased from 3D-Fuel (Donegal, Ireland); (ii) PVA filament was purchased from 3D-Filaprint (Essex, UK), (iii) PLA filament was obtained from 3D-Filaprint (Essex, UK) and iv) Filaflex 82A [14] made of highly flexible thermoplastic polyurethane (TPU), which is a multi-block copolymer consisting of a polyol (soft block) and hard block made of diisocynate and a short chain diol, was obtained from Recreus (Alicante, Spain). Ethanol (96% v/v) and ethyl acetate (ACS grade) were supplied from Alcoholes Aroca S.L (Madrid, Spain) and Panreac (Madrid, Spain) respectively. Humidity capsules, data loggers, and stability chambers were provided by Cuspor Limited^TM^ (Dublin, Ireland). Sodium lauryl sulphate was obtained from Fagron Iberica (Spain, Madrid). All other chemicals employed in the preparation of the buffers were of ACS reagent grade and were used as supplied from Panreac (Madrid, Spain). Solvents were of HPLC grade and were purchased from Scharlab (Madrid, Spain).

### 2.2. Methods

#### 2.2.1. Diffusion Kinetic Studies

A 1 cm in length filament (1.75 ± 0.5 mm of diameter) was immersed in 1 mL of saturated ethanolic solution containing NFD (31.6 mg/mL). The solutions were continuously stirred while being protected from light to avoid NFD photo-degradation at room temperature [15]. At different time points (2, 4, 8, and 24 h), 1 cm length filament sections were withdrawn, dried with tissue paper and placed in an oven at 40 °C for 12 h to ensure solvent evaporation. Afterwards, the amount of NFD that had diffused inside each filament was extracted using a fresh ethanol (2 sets × 2 mL, 2 h each). The solution was then centrifuged (Hettich, Universal 32, Granada, Spain) for five minutes at 5000 rpm and NFD in the supernatant was quantified by UV-spectrophotometer (JASCO, V-730, Tokyo, Japan) at 238 nm. A calibration curve between 0.8 to 50 µg/mL was utilised to determine the amount of NFD in each filament section. The same experiment was repeated using a saturated solution of NFD in ethyl acetate (71.2 mg/mL). The solubility of NFD in both solvents was calculated by UV spectrophotometry. All experiments were performed in triplicate.

The cumulative amounts of NFD diffused into the different filaments were plotted as a function of time [16]. Regression analysis was used to calculate the slopes and intercepts of the linear portion of each graph [17]. The following equation (Equation (1)) was applied to each condition to calculate the steady-state flux:

(1) $$Jss=\frac{dM}{S\times dt\;}$$

where *Jss* is the steady-state flux (mg/cm^2^/h), *dM*/*dt* is the amount of NFD diffused over time into the filament (mg/h), and *S* is the surface area of the filament [18]. Each filament section had the following dimensions: 10 mm in length and 1.75 ± 0.5 mm in diameter, resulting in a total surface area, *S*, of 0.604 ± 0.024 cm^2^, calculated using Equation (2):

(2) $$S=2\pi r^{2}+2\pi rh$$

The permeability coefficient (*P*) into the filaments was calculated by using Equation (3):

(3) $$P=\frac{Jss}{Csat}$$

where *Csat* is the NFD’s saturation solubility in the vehicle (being 31.6 mg/cm^3^ in ethanol and 71.2 mg/cm^3^ for ethyl acetate) [19,20]. Enhancement ratio (ER) was calculated as the ratio of steady-state flux from each filament at earlier times in ethanol compared to ethyl acetate.

#### 2.2.2. Diffusion Kinetics, Mathematical Modelling

In order to investigate the diffusion kinetics of NDF from the solvent into the different filaments, the degree of miscibility between the drug and the solvent molecules and also between the solvent molecule and the filament were calculated. Several approaches, such as the Hansen Solubility theory, have been used in the past to estimate the degree of miscibility of different materials, for example, during co-crystal formation or amorphous solid dispersion development [21,22]. Typically, it is assumed that materials with similar cohesive energy density will be miscible with one another, and the total cohesive energy can be divided into the individual, so-called, Hansen Solubility Parameters (HSP): dispersion forces (*δd*), polar forces (*δp*) and hydrogen bonding forces (*δh*). The values were determined using the van Krevelen group contribution method [23,24], and the total HSP was calculated as follows in Equation (4):

(4) $$\delta t=\sqrt{{\left(\delta d\right)}^{2}+{\left(\delta p\right)}^{2}+{\left(\delta h\right)}^{2}}$$

When representing the three HSP (*δd*, *δp*, *δh*) with their x, y, and z coordinates respectively, a sphere can be plotted in three dimensional space, known as the Hansen sphere of radius, Ro (where Ro is the interaction radius that represents the unique characteristics of each compound). The miscibility between different components can therefore be determined using the Hansen sphere. The distance between coordinates of the target compound (centre of mass of the Hansen sphere represented by *δd*1, *δp*1, *δh*1) and those of the tested compound (*δd*2, *δp*2, *δh*2) is represented by the HSP distance. The HSP distance (*Ra*) between NFD, solvents (ethanol and ethyl acetate selected for their low toxicity and Food Grade suitability), and filaments (PVA, PLA, and HS) was calculated in order to predict their miscibility (Equation (5)); the smaller the Ra value, the larger the miscibility of two components [25,26].

(5) $$Ra=\sqrt{4{\left(\delta d1-\delta d2\right)}^{2}+{\left(\delta p1-\delta p2\right)}^{2}+{\left(\delta h1-\delta h2\right)}^{2}}$$

#### 2.2.3. Optimisation of Filament Drug Loading

The optimum combinations of filament material, solvent, and contact time were selected from the diffusion studies. Selected filaments (PVA and HS) with the highest drug loading (70 cm in length) were immersed in a saturated solution in ethanol (100 mL) containing NFD (31.6 mg/mL) for 8 h at room temperature, protected from light and under gently stirring. After loading, filaments were dried in the oven at 40 °C for 12 h to ensure solvent removal and avoid hydration, prior to printing. Due to loss in rigidity, HS filaments were also left at 100 °C for one hour to enhance their stiffness, which was necessary for printing.

#### 2.2.4. Tablet Design and 3D Printing

##### Geometric Design

The three-dimensional (3D) designs and geometry of the printed tablets were obtained using TinkerCad software (Autodesk 2019, Barcelona, Spain). This tool allows virtual 3D construction of shapes in .stl format. Bearing in mind the density of the filaments (1.25 ± 0.2 g/cm^3^) and the drug loading in each filament, rounded flat tablets were designed to contain 15 mg of NFD with a cylindrical shape. The dimensions for the PVA and HS tablets were 12 × 4.8 mm and 12 × 4 mm, respectively. A combined PVA-HS tablet was also designed consisting of two cylindrical sections of 12 × 2.4 mm (from PVA) and 12 × 2 mm (for HS). Blank tablets using the commercially available filaments, as purchased, were also printed for comparison purposes.

##### Slicing and 3D Printing

Once the design was complete in .stl format, it was uploaded onto a slicing software (Flashprint, Flashforge, Zhejiang Flashforge 3D Technology Co, Zhejiang, China), by which the 3D model was segmented into multiple consecutive layers (38 for PVA tablet and 32 for HS tablet) and transformed into a .X3G code file that the printer can interpret. The printing settings for all the printed tablets were: extrusion temperature of 226 °C, platform temperature of 50 °C, layer height of 0.12 mm apart from the first layer, for which a height of 0.20 mm was selected to ensure good adhesion to the platform, infill of 100% (i.e., no pores were left in the core) and a printing speed of 10 mm/s. A Flashforge Creator Pro (Zhejiang, China) with dual extrusion heads printer was utilised. A single extrusion head was utilised for those tablets containing a single filament material. The combined tablets were printed using both extruder heads in which the PVA and HS filaments were loaded separately.

#### 2.2.5. Physico-Chemical Characterisation of Filaments and Tablets

##### Imaging

To evaluate the dimensions, shape, and surface morphology of the printed tablets, a digital microscope (U500X, CoolingTech, Shenzhen, China) was used and images were processed by ImageJ v1.46 image analysis software (University of Wisconsin, Madison, WI, USA). To gather a more in depth analysis of the morphology of the tablets and filaments at different stages of the experimental process, a Scanning Electron Microscope (Jeol Ltd., Tokyo, Japan) equipped with a secondary electron detector at 15 kV was used after the samples were sputter coated with pure gold using a metaliser (Q150RS Metalizador QUORUM, Quorum Technologies Ltd., Lewes, UK) for 180 s under vacuum [27].

##### Surface Roughness

The surface roughness of the filaments was measured using a Mitutoyo SV-C3200 instrument (Andover, UK). The measured length was 8 mm at a speed of 2 mm/s. The z axis range was set at 800 µm. The arithmetic average roughness (*Rav*) was calculated using the following Equation (6):

(6) $$Rav=\frac{\sum_{n=1}^{N}\left|Zn-Z\right|}{N}$$

where *Zn* is the individual height value of the measurement point by the laser reflection measurement, *Z* is the mean value of all of the height data points, and *N* is the number of measurement point [28]. The absolute vertical distance between the maximum peak height and the maximum valley depth along the sampling length was expressed as Rz.

##### Flexural Strength and Filament Toughness

The mechanical properties of both empty and drug-loaded filaments were evaluated using a texture analyser (Texture Analyser TA-XTplus, Stable Microsystems, Godalming, UK) equipped with a 3-point bend probe manufactured in house (Figure S1, Supplementary Material) [29]. For flexural strength and elongation of the filaments, the 3D printed bend probe set was connected to the load cell. A filament (50 mm, 1.75 mm Ø) was placed on a sample holder with a 25 mm gap. The moving probe reached the surface of the filament with a pre-test speed of 2 mm/s, test speed of 1 mm/s for 10 s and post-test speed of 10 mm/s. The force applied had a trigger load of 4.9 N and the maximum force (mN) and travel distance (mm) were measured and the area under the curve (mN mm) was calculated. Each filament was assessed in triplicate. Exponent software version 6.1.5.0 (Stable Micro Systems, Godalming, UK) was used for data collection and analysis. The bending modulus was calculated as the gradient of the initial linear section of the force-travel distance graph (N.mm). The toughness was considered as the total area under the curve force-travel distance [29].

##### Powder X Ray Diffraction (PRXD)

Full tablets and a cross section of the filaments were loaded in an open holder. A Miniflex II Rigaku diffractometer (Rigaku, Tokyo, Japan) with Ni-filtered Cu Kα radiation (1.54 Å) was used to performed PXRD analysis using a tube voltage of 30 KV and tube current of 25 mA The PXRD patterns were recorded (n = 3) from 5° to 40° on the 2 theta at a step scan rate of 0.05° per second [30].

##### Modulated Temperature DSC (MTDSC)

Modulated temperature DSC (MTDSC) scans were recorded on a QA-200 TA instrument (TA instruments, Elstree, United Kingdom) calorimeter using nitrogen as the purge gas. A section of the tablet and cross section of the filament were weighed (4–6 mg) into non-hermetically closed aluminium pans. A scanning rate of 5 °C/min, amplitude of modulation of 0.796 °C and modulation frequency of 1/60 Hz were employed. The temperature range was between 10 °C and 200 °C [30]. Calibration of the instrument was carried out using indium as standard. Glass transition temperatures reported (n = 3) refer to the midpoint of the transition.

##### Thermogravimetric Analysis (TGA)

Thermogravimetric analysis (TGA) was performed using a Q-50 TA instrument (TA instruments, Elstree, UK). Samples were placed in open aluminum pans (5 mg) and analysed at a constant heating rate of 10 °C/min over a temperature range between 25 °C and 300 °C [30].

##### Near-Infrared (NIR) Spectroscopy

Near-infrared (NIR) analysis was performed using a Luminar 5030 Spectrometer (Brimrose, Tecnilab, Madrid, Spain). A wavelength of 1100–2300 nm was used and 10 average scans with a detector gain of 2 (except when analysing pure NFD power, in which case a detector gain of 1 was undertaken).

##### Dynamic Vapour Sorption (DVS)

Water sorption kinetic profiles of NFD-loaded PVA and HS 3DP tablets were obtained using dynamic vapour sorption (DVS) (Advantage, Surface Measurement Systems, Alperton, UK) at 25.0 ± 0.1 °C. Water was used as the probe vapour. Samples were dried at 0% relative humidity (RH) for 1 h and then subjected to step changes of 10% RH up to 90% RH, and the reverse for desorption. The sample mass was allowed to reach equilibrium, defined as dm/dt ≤ 0.002 mg/min over 10 min, before the RH was changed [30].

##### Friability and Hardness

The 3DP tablets were weighed and then subjected to a friability test according to the USP Pharmacopeia requirement (*n* = 10). A friability apparatus (Pharma Test, PTF, Hainburg, Germany) at 25 rpm (rotations per minute) for four minutes was used. Thereafter, tablets were dusted with a brush and re-weighed. Percentage weight loss was then calculated. 3DP tablets were placed in a tablet hardness testing instrument (Pharma test PTB 311, Germany) and force (N) was applied until tablets cracked [31,32].

##### Uniformity of Content and HPLC Analysis

Drug loading in tablets was assessed in triplicate. Each tablet was added into 100 mL of mobile phase and were bath sonicated for 10 min. All the samples were diluted in mobile phase prior to quantification by HPLC. A Jasco PU-1580 pump attached to a Jasco AS-2050 Plus autosampler and a Jasco UV-1575 UV-visible detector was used (Jasco, Inc., Madrid, Spain). NFD was separated on a Thermo BDS Hypersil C18 reverse phase column (200 × 4.6 mm, 5 µm) (Thermo Fisher Scientific, Madrid, Spain). The injection volume was set at 20 µl and the mobile phase was pumped at 1 mL/min and consisted of methanol: water: acetonitrile (36:55:9, V:V:V). The eluent was monitored at 240 nm. The method showed a liner correlation between drug concentration and absorbance from 50 to 0.5 µg/mL. The detection limit was 0.12 µg/mL while the quantification limit was 0.4 µg/mL.

#### 2.2.6. Dissolution Studies

Dissolution tests were carried out on all types of 3DP tablets (NFD-loaded HS, NFD-loaded PVA and NFD-loaded PVA and HS combined tablets) and Adalat Oros 30 mg modified release tablets, using a type dissolution II apparatus (rotating paddle, at 100 rpm) with six glass 900 mL vessels (DT-80, Erweka, Germany) at 37 °C. Simulated gastric fluid (SGF) with no pepsin (pH 1.2 ± 0.1, 500 mL) and 0.5% w/v sodium lauryl sulphate was utilised for the first 2 h followed by the addition of 400 mL of simulated intestinal medium (with no enzymes, pH adjusted to 6.8 ± 0.1 with 7 mL of NaOH 30% w/v) also containing 0.5% w/v of sodium lauryl sulphate, after which time dissolution testing was continued for a further 22 h, as described in the USP [33,34]. Samples (2 mL) were collected at various time intervals (5, 10, 15, 20, 30 min and at 1, 2, 3, 4, 6, and 24 h), filtered (hydrophilic PTFE 0.45 μm syringe filter, Millipore, Millex-LCR, Madrid, Spain) and diluted (1:3 v/v) with mobile phase prior to HPLC analysis.

##### Mechanistic Mathematical Models

In order to investigate the release from the different tablets, the dissolution data were fitted using the following kinetic equations [35,36]: zero order Equation (7), first order Equation (8), Hixson–Crowell Equation (9) and Korsmeyer–Peppas Equation (10):

(7) $$Q_{t}=K_{0}t$$

(8) $$\ln Q_{t}=\ln Q_{0}+K_{1}t$$

(9) $$Q_{\infty }^{1/3}-{\left(Q_{\infty }-Q_{t}\right)}^{1/3}=K_{s}t$$

(10) $$\frac{Q_{t}}{Q_{\infty }}=K_{KP}t^{n}$$

where *Q_t_* is the amount of drug dissolved in time *t*, *Q*_0_ is the initial amount of drug in the solution (most times, *Q*_0_ = 0), *Q∞* is the initial amount of drug in the tablet (15 mg for 3DP tablets and 30 mg for commercial tablets); *K*_0_ is the zero order release constant; *K*_1_ is the first order release constant, *K_s_* is a constant incorporating the surface-volume relation; *K_KP_* is a constant that describes the structural and geometric characteristics of the drug dosage form; n is the release exponent which describes the drug release mechanism, and it can have a value of 0.5, 0.45 or 0.43 when the particle shape is a thin film, a cylinder or a sphere respectively, which corresponds to Fickian release controlled by diffusion. Anomalous non-Fickian transport is described when n is between those values and 1 (0.5 < *n* < 1 for thin films, 0.45 < *n*< 1 for a cylinder and 0.43 < *n* < 1 for spheres). When *n* = 1, release corresponds to zero order [37]. To test the applicability of the drug release models, the regression coefficient (R^2^) was used [35].

#### 2.2.7. Accelerated Stability Studies

NFD-HS tablets were selected to perform further stability studies. Samples were placed in stability chambers and were exposed to six different conditions of temperature and relative humidity (RH) (Table 1). The temperature was regulated (Memmert VO200 cool, Schwabach, Germany) and the relative humidity was established with different saturated salt solutions or silica [38]. A temperature and RH controller were introduced into the stability chambers to monitor that the appropriate conditions were kept throughout the study. At different time points, samples were withdrawn from the stability chambers and NFD content was analysed by the HPLC method described above. Assessment of stability was based on a change in the nifedipine peak area in the HPLC. Modelling of the stability was based on the humidity-corrected Arrhenius equation (Equation (11)):

Ln(k) = Ln(A) − Ea/RT + B (RH)(11)

where k is the degradation rate (% remaining drug/day), T is the temperature in Kelvin degrees, A is the collision factor, B is the humidity factor, RH is the relative humidity, Ea is the activation energy in kcal mol^−1^, and R is the gas constant (0.00198 kcal K^−1^ mol^−1^) [30].

In order to calculate the degradation rate, the amount degraded at different time points was fitted at each condition to the following kinetic models: zero-order, first-order, second order, Avrami and diffusion using the following equations (Equations (12)–(16)) [39]:

Zero order: [NFD] = kt(12)

1st order: [NFD] = [NFD]_∞_[1−e^(−kt)^] (13)

2nd order: [NFD] = [NFD]_∞_ kt/(1/[NFD]_∞_ + kt) (14)

Diffusion: [NFD] = kt^(1/2)^ (15)

Avrami: [NFD] = [NFD]_∞_ − e^(−kt^2)^ (16)

where t is the time expressed in days, k is the degradation rate of NFD over time, and [NFD] is the amount of NFD expressed in percentage remaining at each time point.

To test the suitability of the models, the regression coefficient (R^2^) was used. The Arrhenius equation was employed to estimate the activation energy and the effect of temperature on the degradation rate of NFD. The degradation constant at 25 °C and 60% RH was extrapolated from the Arrhenius equation (for the HS NFD-loaded 3DP tablets) in order to predict the long-term stability.

#### 2.2.8. Statistical Analysis

Statistical analysis was performed with a one-way ANOVA test followed by Tukey’s test at the 5% statistical significance level using Minitab 16 (Minitab Ltd., Coventry, UK). Linear regression analysis was performed using the method of least squares with Microsoft^®^ Excel software. The Unscrambler^®^ X software (CAMO Analytics Software, Oslo, Norway) was used to perform multivariate analysis. Six variables: (i) HSP distance between each solvent and filament material (Ra); (ii) surface roughness of the filaments, (iii) filament bending modulus; (iv) filament stiffness, (v) steady-state flux of NFD at earlier time points and (vi) permeability were analysed using Principal Component Analysis (PCA). PCA was employed to study the systematic variability and the relationship between variables and scores. The correlation loadings of the principal components (PCs) were represented to understand the variance for each variable for a given PC, giving information about the source of the variability within the dataset [40]. Multiple linear regression, partial least square regression, and support vector machine regression (SVM) models were developed to understand the relationship between Ra, filament surface roughness, bending modulus and stiffness with the steady state flux of NFD into each filament. The Singular Value Decomposition, the NIPALS algorithm, and the radial basic functions were applied to estimate regression and SVM coefficients and PCA calculations. The Root-Mean Square Error (RMSE) was calculated to estimate the goodness of fit of the models [41].

## 3. Results

### 3.1. Diffusion Kinetic Studies and Physicochemical Characterisation

**Figure 1:**
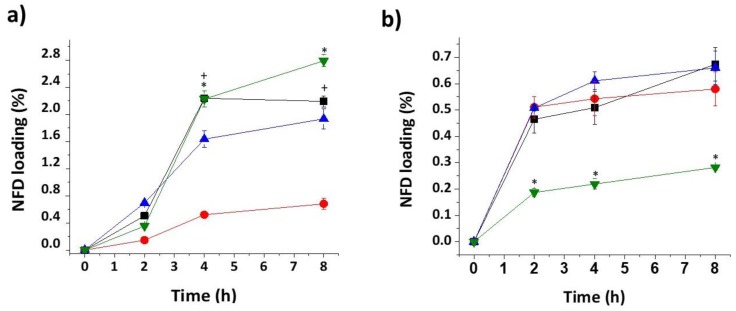
Nifedipine (NFD) loading diffusion kinetics. (**a**) Ethanol, (**b**) Ethyl acetate. Key: Hydrosupport (HS) (green), PVA (black), poly-lactic acid (PLA) (red), and thermoplastic polyurethane (TPU) (blue).

NFD diffusion varied according to the type of filament and solvent utilised to solubilise the drug (Figure 1). The amount of NFD that diffused inside the filament increased sharply within 2 and 4 h, in ethyl acetate and ethanol respectively, followed by a gradual increase until 8 h. Prolonged immersion times (24 h) did not result in greater drug loading (data not shown), which can be explained by the poor stability of NFD in solution [42]. PVA derived filaments (PVA and HS) showed a much greater drug diffusion into the filament for the drug dissolved in ethanol while those made of PLA or TPU exhibited a greater drug diffusion when the drug was dissolved in ethyl acetate. In terms of toughness of the filament, less stiff materials such as TPU exhibited enhanced diffusion kinetic rates compared to stiffer materials, such as PLA. The characteristic behaviour of TPU is conferred by its chemical structure consisting of a hard block (built out of a short-chain diol and diisocyanate) responsible for the toughness and a soft block, constructed from a polyol, responsible for its flexibility [14].

A steeper concentration gradient is expected to enhance passive diffusion. However, although the drug solubility in ethyl acetate was 2.3-fold greater than in ethanol (72 mg/mL vs. 31.6 mg/mL respectively), the NFD diffusion rate into filaments was lower when using ethyl acetate (Figure 1). The enhancement ratio, calculated as the ratio of Jss in ethanol compared to ethyl acetate was 1.6, 2.4, and 5.9 for TPU, PVA, and HS, respectively. The steady-state flux of NFD (J_0–4 h_) was significantly higher for ethanol solutions for all the filaments, except for PLA (Table 2). PVA-derived filaments (PVA and HS in Table 2) exhibited a similar flux in ethanol which was significantly higher than into TPU filament (Table 2). The permeability and diffusion coefficients followed a similar trend to the Jss.

Hansen Solubility Parameters for solvents, filaments, and NFD were calculated using the van Krevelen contribution method [43], and Ra values determined (Table 3). The smaller the value of Ra, the larger the miscibility [25,26]. Ethanol possesses a much greater Ra with NFD than ethyl acetate (13.7 versus 3.3) and this can explain why NFD has a much larger solubility in ethyl acetate than in ethanol, as the latter is a more polar solvent with a higher capacity to hydrogen bond. In solution, NFD creates hydrophobic and hydrophilic interactions with the solvent molecules. However, greater miscibility with the solvent hampers its diffusion into the filament, as higher energy is required to break the bonds between NFD molecules and solvent molecules. The Ra between the solvent and the filament is also key. The higher the miscibility between the solvent and the filament, the higher the diffusivity of the solvent inside the filament. Ethanol showed a smaller Ra with both PVA-derived filaments (ca. 10), while ethyl acetate showed better miscibility with PLA and TPU filaments. Thus, the best solvent–filament combination for a successful passive diffusion requires an equilibrium in miscibility that has to be good enough to ensure diffusivity of the solvent molecules inside the filament, but not so high as to lead to dissolution of the filament within the solvent. Knowing the NFD diffusion rate into different materials allows for the selection of the optimal solvent–filament combination, enabling printing of tablets with a specific volume that contain a tailored drug loading.

**Figure 2:**
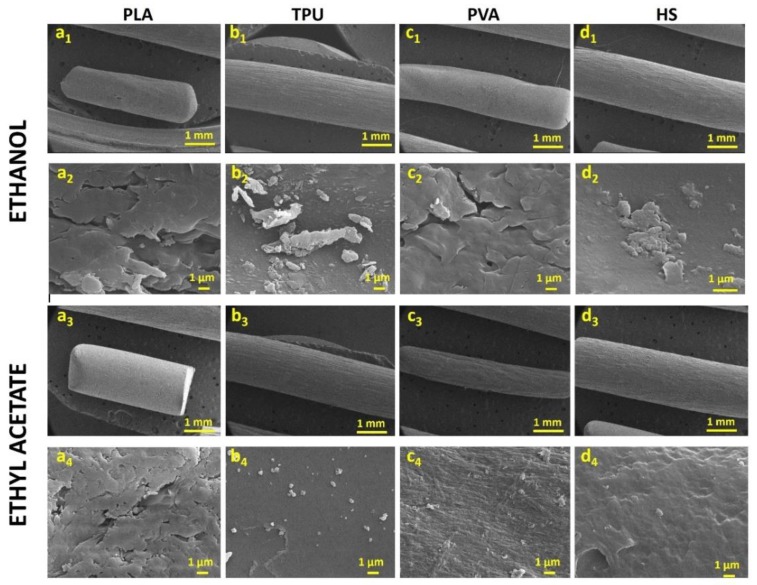
Images obtained by SEM of the four filaments after immersion in ethanol and ethyl acetate for 8 h. Key: (**a1**–**a4**)—PLA, (**b1**–**b4**)—TPU, (**c1**–**c4**)—PVA, and (**d1**–**d4**)—HS. SEM micrographs are shown at two different magnifications.

**Figure 3:**
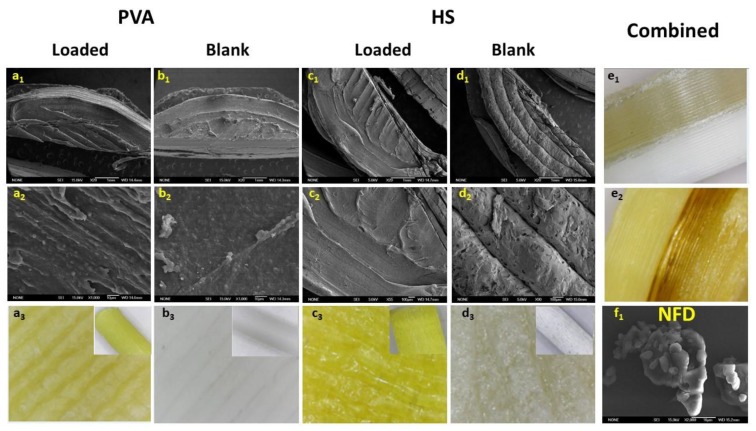
SEM micrographs and digital microscope images of a cut section of the 3DP tablets using PVA and HS NDF-loaded and blank filaments. Key: (**a1**–**a3**)- PVA NFD-loaded tablets; (**b1**–**b3**)- PVA Blank tablets; (**c1**–**c3**)- HS NFD-loaded tablets; (**d1**–**d3**)- HS Blank tablets; (**e1**–**e2**)- Combined 3DP tablets and f1-NFD raw material.

The morphology of the filaments was not affected even after 8 h of immersion in ethanol or ethyl acetate (Figure S2 (Supplementary Information) compared to Figure 2). No major cracks were observed on the surface of any of the tested filaments in the presence of the saturated solution of NFD (Figure 2). However, the formation of micro and nanopores in the filaments over time, which would enhance the passive diffusion of the drug, cannot be excluded. Deposits of small crystals were visible on the surface of the filaments (Figure 2(b2,d2,b4)), especially when ethyl acetate was used as solvent, which can be attributed to NFD that has not fully diffused into the filament and hence, upon drying, crystallises on the surface (Figure 3(f1)).

The best two combinations of filament and solvent, which resulted in highest NFD loading (close to 3% w/w) were HS and PVA in ethanol. Only those filaments were utilised to proceed with the 3D printing of tablets. PVA tablets showed a more opaque colour compared to those tablets printed from HS filaments (Figure 3(a3–d3)). However, the surface of PVA tablets was smoother than HS tablets (Table 3, Figure S3, Supplementary Material). Blank tablets fabricated from the commercial HS filament, in particular, showed a highly porous surface compared to the HS NFD-loaded tablets (Figure 3(d2)). The porosity of PVA prints has previously been shown to be higher than that of PLA prints [45]. Combined tablets consisting of 19 layers of PVA and 16 layers of HS exhibited a brown, darker coloured region in the layer of contact between both polymers, which is likely to be attributable to drug degradation, taking into account that 3D printing occurred at 226 °C, which is close to the degradation temperature of the drug (Figure 3(e2)). Filaments are guided in a PTFE-tube until 3 mm away from extrusion point in the nozzle. The time of exposure inside the extruder nozzle of the printer is quite limited, reducing the risk of drug degradation. However, in order to produce combined tablets of high quality, both extruder heads were loaded at the beginning of the process and combined in situ during the printing. The filament used to print the layers with the second polymer was exposed for a longer period to 226 °C, which could potentially increase the risk of degradation. Nevertheless, the amount of NFD was assayed to be >95% in all the tablets. The problem may be avoided by dispensing a few millimetres of filament to waste prior to start the printing process (priming of the print nozzles). Additionally, a so-called prime tower or skirt could be included in the print.

**Figure 4:**
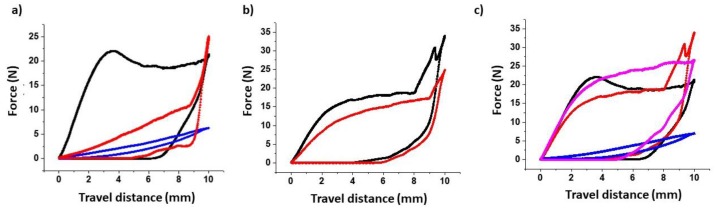
Force-travel distance curve of loaded and blank filaments for 3DP. Measurements were performed in triplicate. Results are expressed as mean ± SD. Key: (**a**) HS filament: blank filament in black, NFD-loaded filament before the drying step in blue and after the additional drying step in red, (**b**) PVA filament: blank filament in black, NFD-loaded filament in red; (**c**) Blank filaments: HS in black, PVA in red, PLA in pink and TPU in blue.

In general, the roughness of both loaded and blank unloaded filaments was significantly smaller compared to the surface of printed tablets (10–15-fold lower). Additionally, the surface roughness of loaded HS filaments was greater than that of blank filaments and has been shown to increase with increased drug loading [5,46]. No major differences were found in the surface roughness between blank filaments. However, the bending modulus and toughness of the filaments were significantly different and were altered during the diffusion step (Table 4). Prior to drug loading, the toughest filament was PLA, followed by HS, PVA, and TPU. Less stiff materials exhibited greater drug diffusion rates, with TPU being the most flexible one. This explains the higher Jss observed for TPU compared to PLA, even though both polymers possess similar HSPs and similar Ra values with the solvents. Chemically, the main difference between PVA and HS filaments is the small fraction of PEG contained in the HS filament. However, a noticeable change occurs in the HS filaments over time, with respect to bending and toughness, after immersion in solvents. Even though the formation of any cracks on the surface of the HS filament it is not evident in the SEM micrographs, its bending and toughness properties were reduced approximately 15-fold after 8 h of immersion in ethanol, while the reduction in stiffness for the PVA filament was minimal when treated in the same manner (Figure 4). This explains why the diffusion of NFD into the HS filament was significantly greater than for the PVA filament after 4 h, despite the two materials having similar HSP values. Loss of stiffness hampers the printing process and has been reported previously by other authors [47]. Thus, the HS needed to be dried for an additional hour at 100 °C prior to printing. After this additional drying step, the bending modulus recovered (2-fold increase) and the stiffness was 5-fold greater (Table 4, Figure 4) enabling successful prints. No drug losses occurred during this process (NFD content >95%).

**Figure 5:**
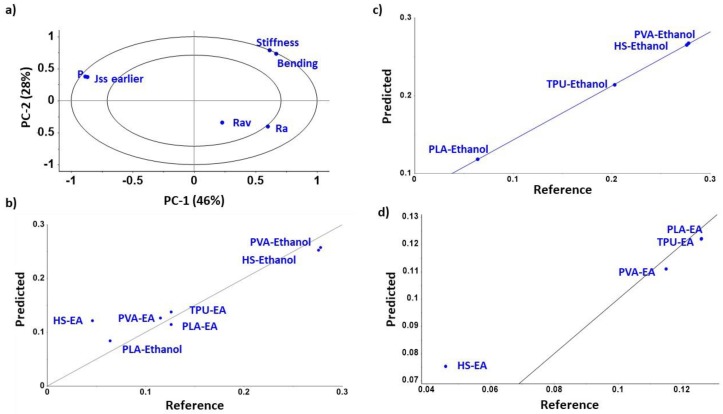
Multivariate statistical analysis. Principal component analysis PC1-PC2 (**a**) and support vector machine regression models of steady state flux into filaments (Jss), including both types of solvents (**b**), just ethanol (**c**), or ethyl acetate (**d**).

In order to develop passive diffusion methods that result in successful drug loading, it is important to understand the main parameters affecting the process and how the final drug loading can be estimated. Our PCA analysis (PC1-PC2 and PC1-PC3) showed a strong inverse correlation between the Jss, permeability, and diffusion with HSP distance (Ra) between solvent and filament and the toughness and bending modulus of the filament (Figure 5A, Figure S4 in Supplementary Material). Hence, the greater the Ra between the filament and the solvent, the lower the diffusivity of the solvent into the filament and hence, the lower the NFD permeability. Additionally, the lower the stiffness and the lower the energy to bend the filament (bending modulus), the greater the diffusion. No correlation was observed for the filament roughness.

Linear regression models (such as multiple linear regression or partial least square regression) showed a poor correlation (R^2^ < 0.5) between the input variables (Ra, roughness and stiffness) and the steady-state flux of NFD into the filaments (Jss). However, support vector machine regression showed a better correlation between the experimental data and the predicted model. When diffusion from each solvent was modelled separately, the R^2^ was greater than 0.98 (Figure 5C,D) while the R^2^ was 0.91 when diffusion data for both solvents was modelled together (Figure 5B). The reason why support vector machine regression works better than linear regression models is because it employs kernel functions to map from the original space to feature space providing the ability to handle nonlinear regression cases. Kernel functions can be viewed as a mapping of nonlinear data to a higher dimensional feature space [48].

### 3.2. Further Physicochemical Characterisation

**Figure 6:**
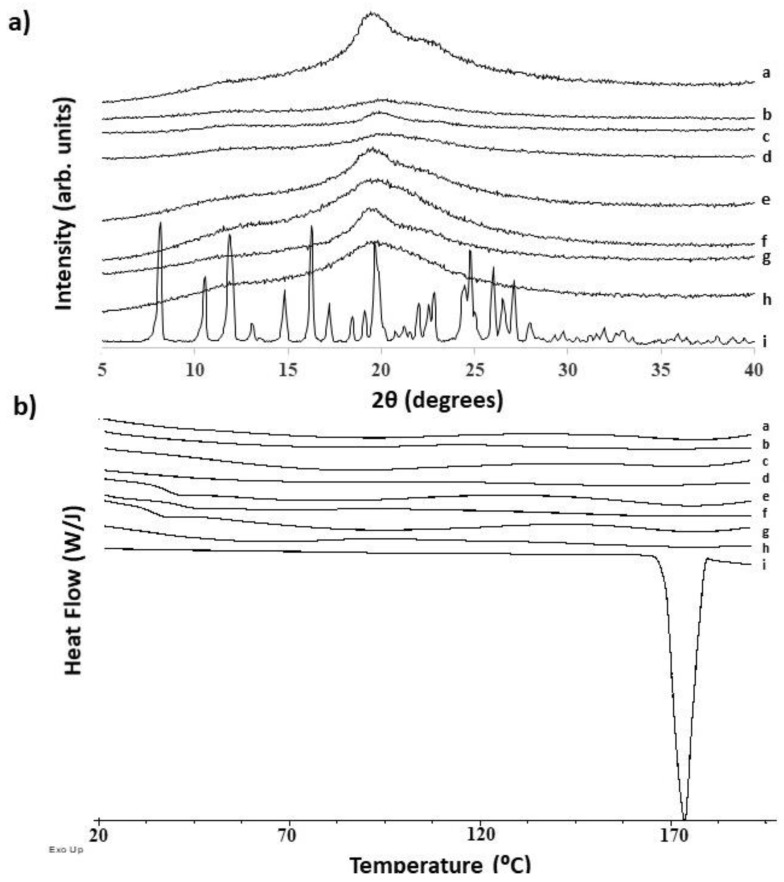
PXRD patters (**a**) and DSC thermograms (**b**) of filaments and 3DP tablets. Key: a—HS NFD-loaded tablet, b—PVA NFD-loaded tablets, c—HS NFD-loaded filament, d—PVA NFD-loaded filament, e—HS blank tablet, f—PVA blank tablet, g—HS blank filament, h—PVA blank filament, i—NFD raw material.

Filaments and 3DP tablets were also characterised by PXRD, DSC, TGA, NIR, and DVS. Figure 6(i) clearly illustrates the crystalline nature of unprocessed NFD exhibiting characteristic Bragg peaks and a sharp melt endotherm with an onset of 168.5 ± 0.2 °C and a heat of fusion of 113.8 ± 0.7 J/g.

No traces of Bragg peaks or endothermic peaks attributed to NFD were found in any of the loaded filaments or 3DP tablets. Both PLA and PVA are semi-crystalline polymers, the crystalline index of which depends on the synthetic process and the physical aging [49,50]. In the diffractograms, a broad peak was observed in the region between 17 and 25 2θ degrees (Figure 6a). No endothermic peak was observed in the region of analysis ranging from 25 to 200 °C in any of the blank or loaded filaments or 3DP tablets (Figure 6b). However, a characteristic glass transition (Tg) was observed in all the systems, except for those containing HS loaded with NFD (Table 5). The reversing signal of the blank HS filament and 3DP tablet exhibited a Tg with a middle point of 34.6 and 36.9 °C respectively (Figure S5 in Supplementary Material). The heat capacity of the HS blank filament and tablet was 3 to 4- fold greater than the equivalent blank PVA systems. Bearing in mind that the immersion in ethanol led to a 15-fold loss in stiffness of the polymer and a 3% w/w loading of drug, the polymeric chains of HS may have suffered from a rearrangement in which the amorphous domains were destabilised. This may be related to dehydration as a result of ethanol evaporation, and removal of necessary water molecules. The ΔCp values at Tg are expected to increase with increasing fragility of the materials. The increase in ΔCp provides a relative measurement of the number of accessible molecular conformations at Tg, giving an indication of the ease of crystallisation [51]. This is supported by the lower resistance to bend (higher flexibility) observed in the three-point bend experiment for the HS polymer compared to PVA filaments.

Blank unprocessed PVA filaments showed a clear Tg at 104.2 °C (Table 5, Figure S5, Supplementary Material) which shifted to lower temperatures once the NFD was loaded (93.97 °C). The 3D printing process had an impact on the thermal history of the samples considering that the loaded polymer is melted and quickly cooled in a matter of seconds. The Tg in the 3DP NFD-loaded tablets was decreased to 89.09 °C, which can be attributed to the miscibility between the drug (with a Tg of 46.2 °C [52]) and the polymer, leading to the formation of a single Tg mix. The most surprising event was the double Tg (at 103.4 and 39.48 °C) observed only in the blank 3DP PVA tablet. Although PVA is often taken to be a homopolymer, the structure of commercial PVA is usually a copolymer of vinyl alcohol and vinyl acetate [53]. During the 3DP process, the amorphous domains of each polymer may be separated and as a result two clear Tg are observed in the thermogram (Figure S5, Supplementary Material). Similarly, to the HS filament, the diffusion of nearly 3% w/w NFD inside the filament may lead to a new rearrangement of polymer chains, which explains the shift of the Tg to lower temperatures.

**Figure 7:**
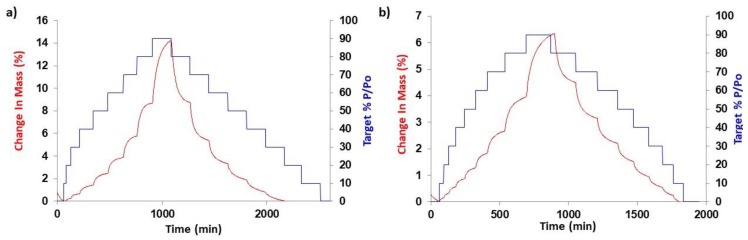
Water sorption kinetic profiles of HS NFD-loaded 3DP tablets (**a**) and PVA NFD-loaded 3DP tablets (**b**).

Regarding the water uptake, HS filaments have greater capacity to sorb water molecules than PVA. Hygroscopicity is more significant upon printing, as the water content increased by 3-fold both in the blank and HS NFD- loaded tablets (Table 4, Figure S6, Supplementary Material). Additionally, the percentage of water loss from HS NFD-loaded tablets was double that of PVA loaded tablets. This result is aligned with the water sorption profile obtained by DVS for PVA and HS NFD loaded tablets (Figure 7). At 90% RH, the amount of water uptake was 14% versus 6% for HS and PVA loaded tablets, respectively. These results suggest that more porous and hydrophilic surfaces are exposed to the environment in the case of HS NFD-loaded tablets.

**Figure 8:**
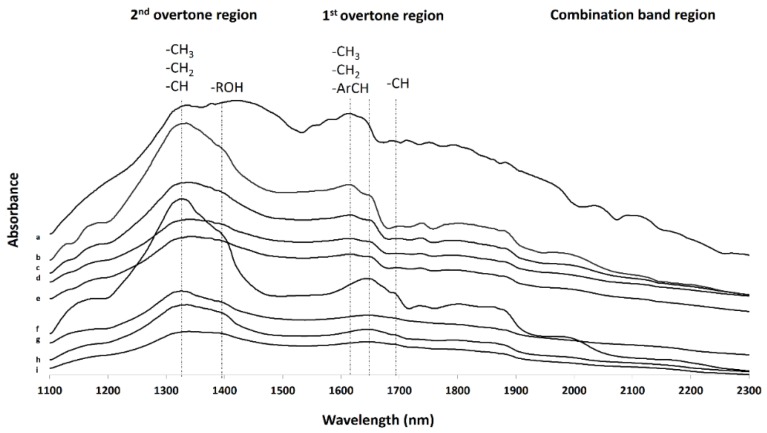
Near-infrared (NIR) spectra of filaments and 3DP tablets. Key: (**a**) Raw material NFD; (**b**) PVA NFD-loaded tablet; (**c**) PVA blank tablet; (**d**) PVA loaded filament; (**e**) PVA blank filament; (**f**) HS NFD-loaded tablet; (**g**) HS blank tablet; (**h**) HS loaded filament; (**i**) HS blank filament.

The NIR spectra of filaments and 3DP tablets are represented in Figure 8. The most characteristic bands are those located in the range from 1296 to 1329 nm and 1619 to 1698 nm, corresponding to the vibrations of –CH3, –CH2, and –CH in the 2nd and 1st overtone region, respectively. The latter bands were shifted towards higher wavelengths in the PVA processed systems but especially in those containing HS polymer. More intense bands were observed in the 3DP loaded tablets which is indicative of hydrophobic interactions between NFD and polymer chains. Additionally, the band for the –ROH was found in the PVA and HS systems at ~1400 nm, being more prominent in NFD-loaded systems than in the blank tablets, which can be attributed to hydrophilic interactions with the drug such as H-bonds.

### 3.3. Dissolution Studies

NFD 3DP printed tablets (15 mg) were prepared based on the filament density and the maximum drug loading in each of the filaments (2.7% w/w and 2.2% w/w for HS and PVA filaments respectively). Manufactured tablets had a theoretical volume of 0.452 cm^3^ and 0.542 cm^3^ (without including air voids between printed lines) and a final tablet weight of 550 ± 15 mg for the HS tablets and 670 ± 13 mg for the PVA tablets, respectively. 3DP tablets exhibited similar dimensions to commercially available tablets (Figure S7. Supplementary Material). The drug loading in each of the tablets complied with the EU Pharmacopeia specification (>95%). HS and PVA tablets also complied with the friability specification, being below 0.1%. In both cases, the hardness of the 3DP tablets surpassed >200 N and, at this force, which is the highest force exerted by the equipment, the tablets did not break.

**Figure 9:**
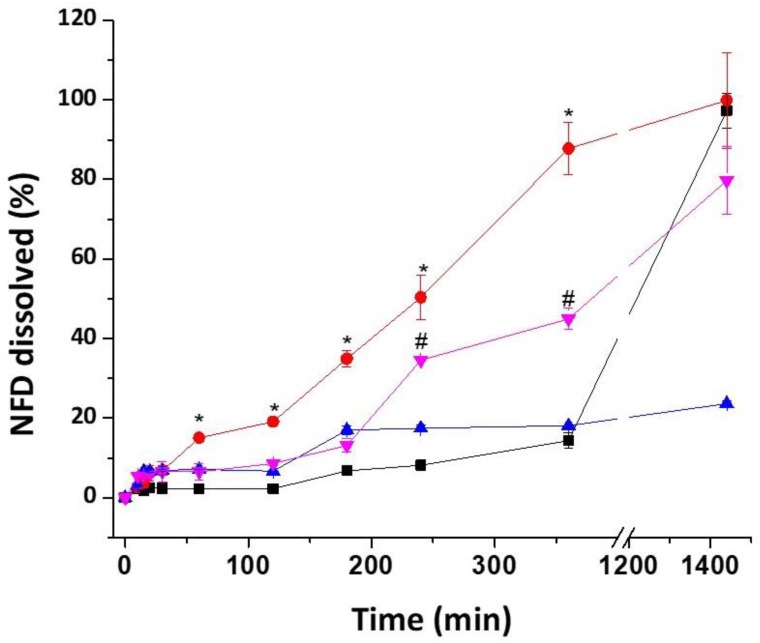
Dissolution profile of 3DP tablets compared to NFD commercially available formulations. Key: HS NFD-loaded tablet (red), PVA NFD-loaded tablet (blue), Combined tablet (pink), Adalat Oros (black). * *p* < 0.05 for HS NFD-loaded tablet, and # *p* < 0.05 for Adalat Oros.

Dissolution studies showed a different release profile for all the tested formulations (Figure 9, Figure S8, Supplementary Material). HS 3DP tablets exhibited the fastest release with 85% drug dissolved at 6 h, which matches with the high water uptake observed by DVS studies. The release of NFD from HS tablets was pH independent, unlike Adalat Oros tablets, which showed a lag time in acidic medium (up to 120 min, Figure S7, Supplementary Material). From 120 min up to 24 h, Adalat Oros tablets showed a zero order release, controlled through the orifice that it is located at the surface of the tablet [54]. In contrast, PVA tablets showed an extremely slow drug release, resulting in approximately 20% drug being released after 24 h. Thus, it was decided to fabricate a combined HS and PVA tablet with fast and slow release segments that would more closely match the profile of the commercially available Adalat Oros. The combined tablet shows better sustained release over a prolonged period of time. The Korsmeyer-Peppas kinetic profile better fitted the release for all three types of 3DP tablets (Table S1, Supplementary Material). During dissolution, the 3DP tablets did not disintegrate. HS tablets and composite tablets eroded from the outer layer (Figure S9, Supplementary Material). In the case of HS tablets, the surface of the tablet formed a gel layer from which the drug was released to the liquid medium. PVA tablets did not show any erosion and their shape remained intact and unaltered over the 24 h of the study. Drug release in this case was driven by diffusion (n = 0.439). Lack of tablet disintegration during dissolution studies has been observed previously by other authors [55]. Drug release is explained by three consecutive steps, diffusion of water into the tablet followed by the solubilization and diffusion of drug through micropore channels within the polymeric matrix.

### 3.4. Stability Studies

As a proof of concept, the chemical stability of the drug within the HS 3DP tablet was compared to NFD raw material (Table 6). The degradation kinetics for the 3DP tablets followed an Avrami-order degradation (R^2^ =0.92), while the decomposition of NFD raw material better fitted zero-order degradation kinetics (Table S2, Supplementary Material). The activation energy for the NFD raw material was greater than for the NFD contained in the 3DP solid dosage form, making the latter more susceptible to high temperatures. The sensitivity to moisture was greater for the 3DP NFD tablet, which can be attributed to the amorphous state of the drug compared to the NFD raw material, which is highly crystalline. The Avrami kinetic model is usually applied to evaluate the growth and the formation of crystals. In this case, the 3DP structure can become heterogeneous over time and as the degradation progresses, the combined effect of physical and chemical degradation can take place simultaneously [39].

From the modified-Arrhenius equation obtained from extreme conditions of temperature and relative humidity, the shelf life at a mean temperature of 25 °C and a mean RH of 60% was predicted. The prediction estimated that it takes over three years for 10% degradation to occur at the above-mentioned conditions in both the raw NFD and the HS 3DP tablet. Appropriate packaging would extend the shelf-life of the 3D printed product even further, bearing in mind that the stability assay was performed on the unpackaged pharmaceutical dosage form. Based on these results, HS NFD-loaded 3DP tablets may be considered to be as stable as commercially available NFD oral dosage forms [56].

## 4. Discussion

This work demonstrates that nifedipine dose can be tailored using passive diffusion and FDM. This can be applied in poly-medicated patients that require a few small doses of several APIs for example, for hypertension and cholesterol. The applicability of personalised FDM 3DP medicines is currently limited by the need to manufacture printable filaments using a hot melt extruder, slowing down the translation of this technique into clinical practice. Only extremely potent drugs are typically used, as passive diffusion results in very low drug loading (<1–2%) in most cases [11,12,13]. One of the reasons is that solvent and filament composition have not been optimized and hence a negligible loading is obtained. Solvent should be carefully selected to ensure that it can diffuse inside the filament.

In this work, the feasibility of passive diffusion reaching a drug loading close to 3% w/w has been demonstrated, enabling the manufacture of solid dosage forms that contain up to 40 mg of drug. Our algorithm based on HSP distances (Ra) between the solvent, the drug, and the filament provides an easy guide to facilitate the drug loading process, and has potential for development into a commercial software. However, the model has been developed with a limited number of samples and replicates and hence, it should be further validated with a larger number of compounds and filament compositions. In order to enhance the drug’s passive diffusion from the solvent to the filament it is necessary to overcome the interactions between drug and solvent molecules in order to allow the drug to diffuse freely inside the solid structure of the filament. Thus, a high HSP distance between the drug and the solvent and a lower HSP distance between the solvent and the filament makes the process energetically more favourable.

This is the first time that a prediction model of passive diffusion into commercially available filaments has been established. The model is based on a support vector machine regression; in addition to the HSP distances (Ra), it also takes into account other material properties such as filament stiffness and flexibility. The HS filament possesses several characteristics that make it optimal for enhanced drug loading via passive diffusion. The commercial HS filament is as tough as PLA and has good printability characteristics. However, after immersion in safe-for-human-consumption solvents, such as ethanol, the polymer chains seem to rearrange themselves, resulting in a significant loss of stiffness; however, an additional short drying step of 1 h was found to be sufficient in order to recover the printable characteristics of the filament.

PVA filaments also showed good drug loading capacity (2.2% w/w), however the release from the polymer matrix when the loaded tablet was immersed in the dissolution medium was incomplete over 24 h. PVA filaments prepared with low molecular weight polymer chains would be preferable in ensuring dissolution of 3DP tablets under mild conditions of temperature (37 °C) and stirring within reasonable timeframes. Finally, it is crucial that filaments are prepared with pharmaceutical grade excipients and are supplied with comprehensive characterisation data that can ensure standardisation of the properties that impact on the quality and safety of the printed dosage forms [6].

3DP, and in particular FDM, can lead to extremely hard tablets when 100% infill is used, which slows down even further the dissolution process [57]; several solutions have been proposed by several authors such as the modification of the layer height during printing, altering the intra-print surface area, and the reduction of the percentage of infill inside the tablet, but the latter can only occur at the expense of loading and thus necessitates a higher volume of the dosage form [58,59]. More hydrophilic inner fills can be also added to speed up the dissolution process [60]. The HS tablets do not require a reduction in the percentage of infill as they erode. Drug dissolution occurs by diffusion from the dense core, and also from erosion of the outer layers, resulting in a 24 h sustained release. An advantage of the HS 3DP tablet compared to commercially available dosage forms is that its release is pH independent and hence, in the case of NFD, a quicker and more reproducible control of blood pressure may be expected. If a more prolonged sustained release profile is desired, this can be easily obtained by printing tablets with combined segments of HS and PVA filaments, as we have demonstrated.

This study is one of the very few existing reports on accelerated predictive stability (APS) testing performed on 3D printed medicines. The long-term stability is not commonly tested in this type of medicine because it is expected that, as they are personalised medicines prepared as extemporaneous formulations, tablets need only be stable for a few weeks or months after manufacturing. The low printing speed for personalised medicines (~10 min tablet) can be increased by increasing the layer height and nozzle diameter, especially when high resolution is not crucial. Our HS tablets have shown a long-term predicted stability of over three years at 25 °C and 60% RH, as the dense core reduces molecular mobility and thus enhances the chemical stability of the formulation. This demonstrates that 3DP medicines can be stored for long periods of time, similar to industrially fabricated solid dosage forms.

## 5. Conclusions

Finding a suitable combination of drug solvent–filament is crucial to ensure the manufacture of 3DP tablets with characteristics similar to those of commercially available solid dosage forms. HSP distance (Ra) values between drug, solvent, and filament can be used as a pre-screening tool to select the most optimal combination, by targeting a high Ra between the solvent and the drug and a Ra value of about 10 between the solvent and the filament. The surface roughness and stiffness of filaments also appear to play key roles in enhancing the passive diffusion of drug into the polymer. HS NFD-loaded tablets have been shown to possess most promising characteristics, with a sustained release over 24 h, long-term stability, and high chemical stability, similar to Adalat Oros tablets. Our study shows that passive diffusion coupled with FDM demonstrate good potential to be implemented in clinical settings to manufacture personalised medicines.

## Figures and Tables

**Table 1:** Conditions and sampling time used for the accelerated stability study. Key: RH, relative humidity.

Environmental Conditions	Sampling Time (Day)	Humidity Control Method
80 °C, 75% RH	14, 28, 48	Saturated sodium chloride aq. soln.
80 °C, 10% RH	14, 28, 48	Silica gel (3–5 mm)
70 °C, 50% RH	14, 28, 48	Saturated sodium bromide aq. soln.
70 °C, 10% RH	14, 28, 48	Silica gel (3–5 mm)
60 °C, 75% RH	14, 28, 48	Saturated sodium chloride aq. soln.
50 °C, 50% RH	14, 28, 48	Saturated sodium bromide aq. soln.

**Table 2:** Diffusion kinetics of NDF from different solutions into different filaments. Key: Jss, steady state flux calculated from the slope of cumulative amount of drug diffused into the filament versus time (mg/cm^2^/h); ER, Enhancement ratio, calculated as the ratio of steady state flux in each filament in ethanol compared to ethyl acetate; P, permeability coefficient (cm/h); - Not applicable.* *p* < 0.05 ethanol versus ethyl acetate.

Solvent	Filament	Jss_0–4h_ (mg/cm^2^/h)	P_0–4h_ (cm/h) × 10^3^	ER
Ethanol	PVA	0.278 * ± 0.065	8.794 * ± 0.741	2.4
	PLA	0.064 ± 0.012	2.044 * ± 0.315	0.5
	TPU	0.203 * ± 0.054	6.424 * ± 0.568	1.6
	HS	0.276 * ± 0.078	8.744 * ± 0.874	5.9
Ethyl acetate	PVA	0.115 ± 0.018	1.615 ± 0.058	-
	PLA	0.126 * ± 0.045	1.771 ± 0.084	-
	TPU	0.126 ± 0.032	1.771 ± 0.077	-
	HS	0.046 ± 0.014	0.652 ± 0.029	-

**Table 3:** Hansen Solubility Parameters (HSP obtained from [44]) for solvents, nifedipine (NFD) and excipients and HSP distance (Ra) values. Key: Dispersion forces (δd), polar forces (δp), and hydrogen bonding forces (δh). NA, not applicable.

Material	Total HSP (MPa^1/2^)	δ_d_ (MPa^1/2^)	δ_p_ (MPa^1/2^)	δ_h_ (MPa^1/2^)	R_a NFD_ (MPa^1/2^)	R_a Ethanol_ (MPa^1/2^)	R_a Ethyl acetate_ (MPa^1/2^)
NDF	18.3	16.6	2.4	7.4	NA	NA	NA
Ethanol	26.5	15.8	8.8	19.4	13.7	NA	NA
Ethyl acetate	18.1	15.8	5.3	7.2	3.3	NA	NA
PVA	28.9	15.0	17.2	17.8	18.4	9.5	13.9
HS	29.0	15.1	16.9	18.1	18.3	9.7	14.1
PLA	21.9	18.6	9.9	6.0	8.6	16.2	21.8
TPU	20.8	18.1	9.3	4.5	8.1	17.8	25.1

**Table 4:** Surface roughness and bending modulus of filaments and 3D printing (3DP) tablets. Key: Rav represents the average roughness values measured over sampling length (arithmetic mean of absolute ordinate values within sampling length), while Rz represents the maximum height of the profile indicating the absolute vertical distance between the maximum peak height and the maximum valley depth along the sampling length. Not determined (-). Values in brackets correspond to the filament data before the drying step. * *p* < 0.05 surface roughness of 3DP tablets versus filaments; # *p* < 0.05 in filament toughness; + *p* < 0.05 in filament bending.

System	Rav (μm)	Rz (μm)	Filament Bending Modulus (N mm)	Filament Toughness—AUC (N mm)
HS blank tablet	29.95 ± 3.40	215.03 ± 27.18	-	-
HS NFD-loaded tablet	32.86 ± 4.12	254.72 ± 45.12	-	-
HS blank filament	2.99 * ± 0.14	15.2 * ± 0.51	7.86 ± 0.33	141.80 ^#^ ± 5.94
HS NFD-loaded filament	3.47 * ± 0.19	26.18 * ± 3.28	1.29 ^+^ ± 0.37 (0.61 ^+^ ± 0.03)	44.11 ^#^ ± 10.62 (8.91 ^#^ ± 1.36)
PVA blank tablet	25.14 ± 3.12	156.24 ± 14.87	-	-
PVA loaded tablet	27.56 ± 2.77	172.95 ± 11.95	-	-
PVA blank filament	1.84 * ± 0.66	13.05 *± 3.54	7.11 ± 1.22	130.82 ^#^ ± 13.50
PVA NFD-loaded filament	2.38 * ± 0.60	15.69 * ± 0.19	3.69 ^+^ ± 0.59 (3.62 ± 0.48)	102.98 ^#^ ± 5.02 (100.45 ± 4.98)
PLA blank filament	2.79 ± 0.08	15.39 ± 0.71	6.84 ± 0.05	157.50 ^#^ ± 2.02
TPU blank filament	2.24 ± 0.12	14.58 ± 0.42	0.72 ^+^ ± 0.05	12.26 ^#^ ± 4.50

**Table 5:** Glass transition temperature (Tg, middle temperature), heat capacity (Cp) and loss of water up to 100 °C of filaments and 3DP tablets. Data is shown as average of three measurements and standard deviation. Key: - not observed. * *p* < 0.05 versus blank filament.

System	Tg (°C)	ΔCp (J/g °C)	Percentage of Water Loss (%)
PVA blank filament	104.20 ± 0.16	0.158 ± 0.007	0.57 ± 0.14
PVA blank tablet	103.4 ± 0.57 39.48 ± 6.26	0.147 ± 0.014 0.194 ± 0.039	0.83 ± 0.21
PVA NFD-loaded filament	93.97 * ± 1.06	0.206 * ± 0.032	0.69 ± 0.11
PVA NFD-loaded tablet	89.09 * ± 0.99	0.206 *± 0.002	1.12 * ± 0.13
HS blank filament	34.63 ± 1.58	0.458 ± 0.004	0.69 ± 0.18
HS blank tablet	36.99 * ± 0.01	0.735 * ± 0.022	2.58 * ± 0.32
HS NFD-loaded filament	-	-	0.78 ± 0.24
HS NFD-loaded tablet	-	-	2.28 * ± 0.31

**Table 6:** Estimated Arrhenius equation parameters using the best fitting kinetic degradation model. Key: Ea: activation energy and B, RH sensitivity value. Shelf life was estimated at 25 °C and 60% RH. The specification limit was set to 10%. Values are reported as mean ± standard deviation.

Parameter	Raw NFD	HS NFD-Loaded 3DP Tablet
Ea (Kcal/mol)	23.38 ± 3.45	10.25 ± 1.64
B	0.008 ± 0.003	0.022 ± 0.008
R^2^	0.894	0.920
Kinetic model	Zero order	Avrami order
Shelf life	>3 years	>3 years

## Supplementary Materials

The following are available online at https://www.mdpi.com/1999-4923/12/4/345/s1. Figure S1. Three-point bed probe utilised with the texture analyser equipment. The 3D probe set was printed from PLA and consisted of a rectangular section of 10 × 5 × 2.5 cm attached to an isosceles right-angle triangle on the top (base: 5 × 2.5 cm, height: 1 cm). Figure S2. SEM micrographs of HS (left) and PVA (right) blank filaments. Figure S3. Images of 3DP tablets, empty and NFD-loaded, printed from PVA and HS filaments. Figure S4. PC1 versus PC3. Figure S5. TM-DSC thermograms (reversing signal) of filaments and 3D printed tablets. Key: a- HS NFD-loaded tablet, b- HS NFD-loaded filament, c- HS blank tablet, d-HS blank filament, e-PVA NFD-loaded tablets, f- PVA NFD-loaded filament, g- PVA blank tablet, h- PVA blank filament. Figure S6. Thermogravimetric analysis results obtained from filaments and 3DP tablets. Key: (a) PVA blank tablet; (b) PVA blank filament; (c) PVA NFD-loaded tablet; (d) PVA NFD-loaded filament; (e) HS blank tablet, (f) HS blank filament; (g) HS NFD-loaded tablet; (h) HS NFD-loaded filament. Figure S7. Size of 3DP tablets compared to Adalat Oros tablet. Figure S8. Dissolution profile of 3DP tablets compared to NFD commercially available formulations. Key: HS NFD-loaded tablet (red), PVA NFD-loaded tablet (blue), Combined tablet (pink), Adalat Oros (black). Table S1. R^2^ data for different kinetic dissolution models (1st 6 h). Figure S9. Hybrid tablet during the dissolution study (after 6 h). Top surface corresponds to HS section and bottom part corresponds to PVA section. Table S2. R^2^ data for different kinetic stability models.
