# Supplementary material for: Personalised 3D Printed Medicines: Optimising Material Properties for Successful Passive Diffusion Loading of Filaments for Fused Deposition Modelling of Solid Dosage Forms
Source: Pharmaceutics. 2020 Apr 11;12(4):345. doi: 10.3390/pharmaceutics12040345 (PMC7238181; doi:10.3390/pharmaceutics12040345)
Supplement: Supplementary file 1 [file pharmaceutics-12-00345-s001.pdf]

pharmaceutics

MDPI

Supplementary Material

Personalised 3D Printed Medicines: Optimising Material Properties for Successful Passive Diffusion Loading of Filaments for Fused Deposition Modelling of Solid Dosage Forms

Jose R. Cerda [1], Talaya Arifi [2], Sejad Ayyoubi [1], Peter Knief [3], Maria Paloma Ballesteros [1], William Keeble [4], Eugen Barbu [2], Anne Marie Healy [5], Aikaterini Lalatsa [2] and Dolores R. Serrano[1,*]

[1] Departament of Pharmaceutics and Food Technology and Instituto Universitario de Farmacia Industrial (IUFI), School of Pharmacy, University Complutense, Avenida Complutense, 28040 Madrid, Spain; jcerda@ucm.es (J.R.C.); sejadayy@gmail.com (S.A.); pballesp@ucm.es (M.P.B.)

[2] Biomaterials, Bio-engineering and Nanomedicine (BioN) Lab, Institute of Biomedical and Biomolecular Sciences, School of Pharmacy and Biomedical Sciences, University of Portsmouth, White Swan Road, Portsmouth PO1 2 DT, UK; talaya.arifi@myport.ac.uk (T.A.); eugen.barbu@port.ac.uk (E.B.); katerina.lalatsa@port.ac.uk (A.L.)

[3] UCD Centre for Precision Surgery, Catherine McAuley Education and Research Centre, Dublin 7, Ireland; peter@knief.de

[4] Faculty of Technology, University of Portsmouth, White Swan Road, Portsmouth PO1 2 DT, UK; william.keeble@port.ac.uk

[5] SSPC The SFI Research Centre for Pharmaceuticals, School of Pharmacy and Pharmaceutical Sciences, Trinity College Dublin, Dublin 2, Ireland; healyam@tcd.ie

* Correspondence: drserran@ucm.es; Tel.: +34-91-394-1620

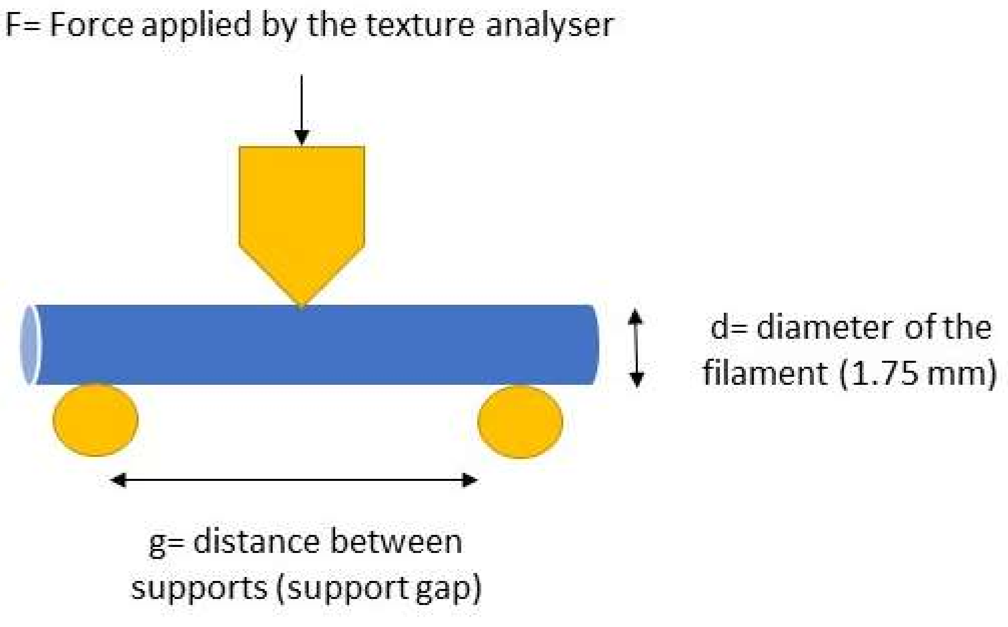

Figure S1. Three-point bed probe utilised with the texture analyser equipment. The 3D probe set was printed from PLA and consisted of a rectangular section of 10 x 5 x 2.5 cm attached to an isosceles right-angle triangle on the top (base: 5 x 2.5 cm, height: 1 cm).

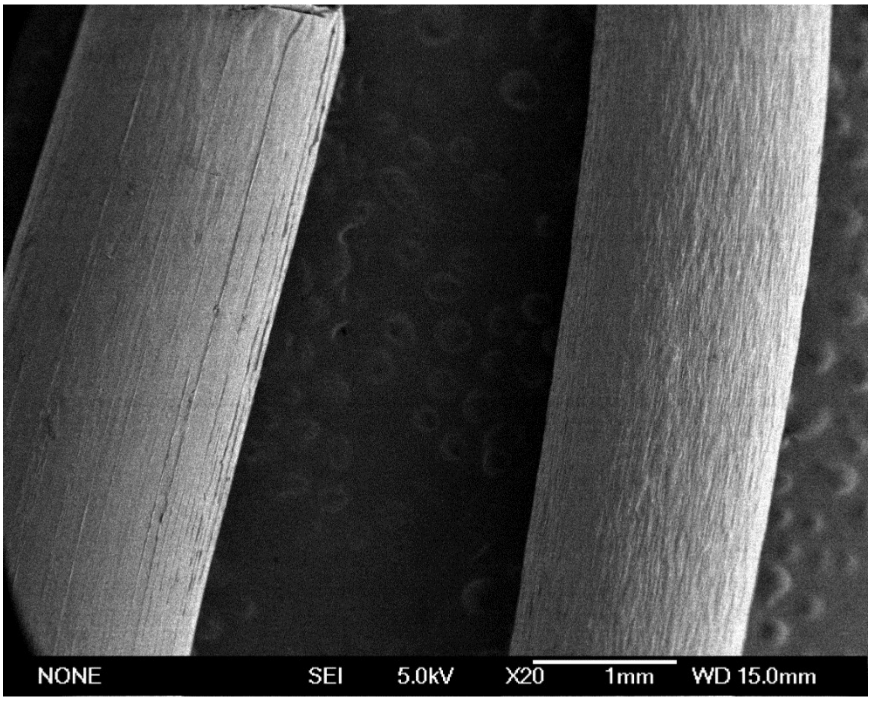

Figure S2. SEM micrographs of HS (left) and PVA (right) blank filaments.

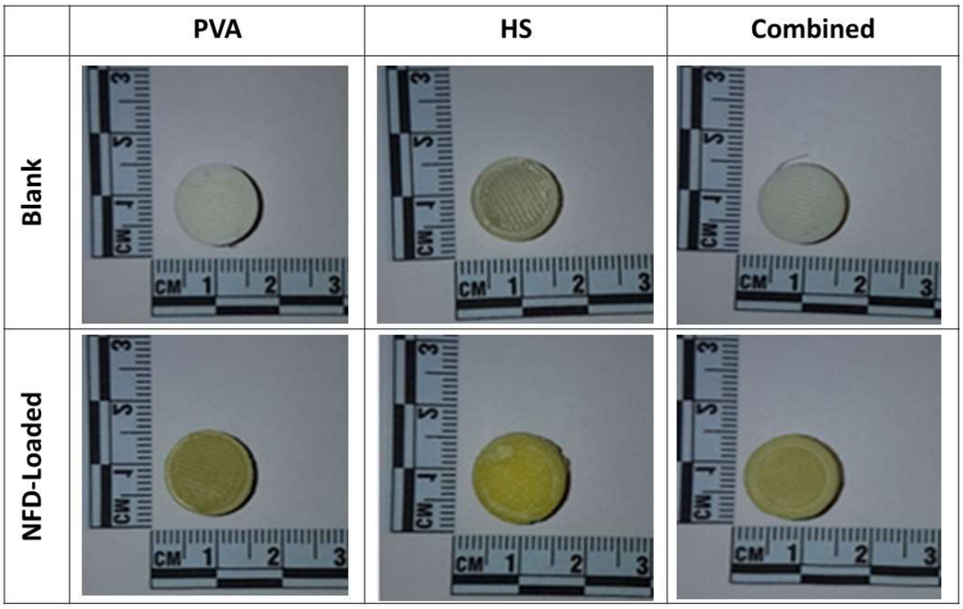

Figure S3. Images of 3DP tablets, empty and NFD-loaded, printed from PVA and HS filaments.

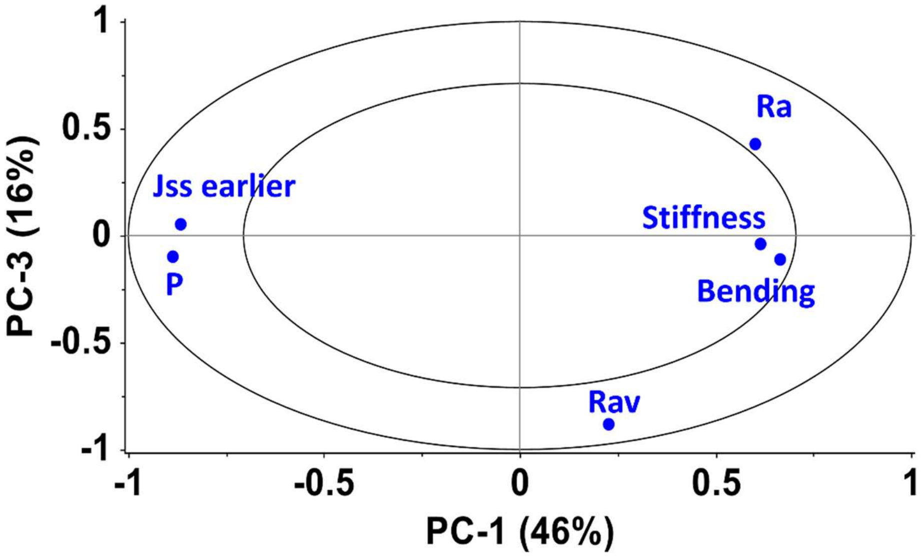

Figure S4. PC1 versus PC3.

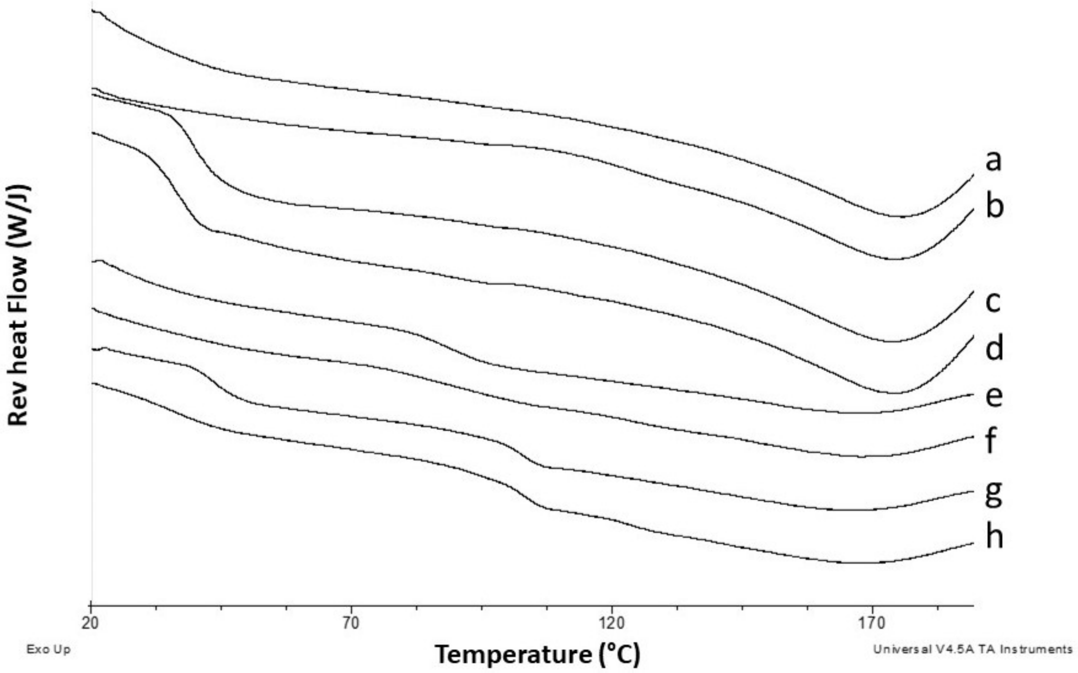

Figure S5. TM-DSC thermograms (reversing signal) of filaments and 3D printed tablets. Key: a- HS NFD-loaded tablet, b- HS NFD-loaded filament, c- HS blank tablet, d-HS blank filament, e-PVA NFD-loaded tablets, f- PVA NFD-loaded filament, g- PVA blank tablet, h- PVA blank filament.

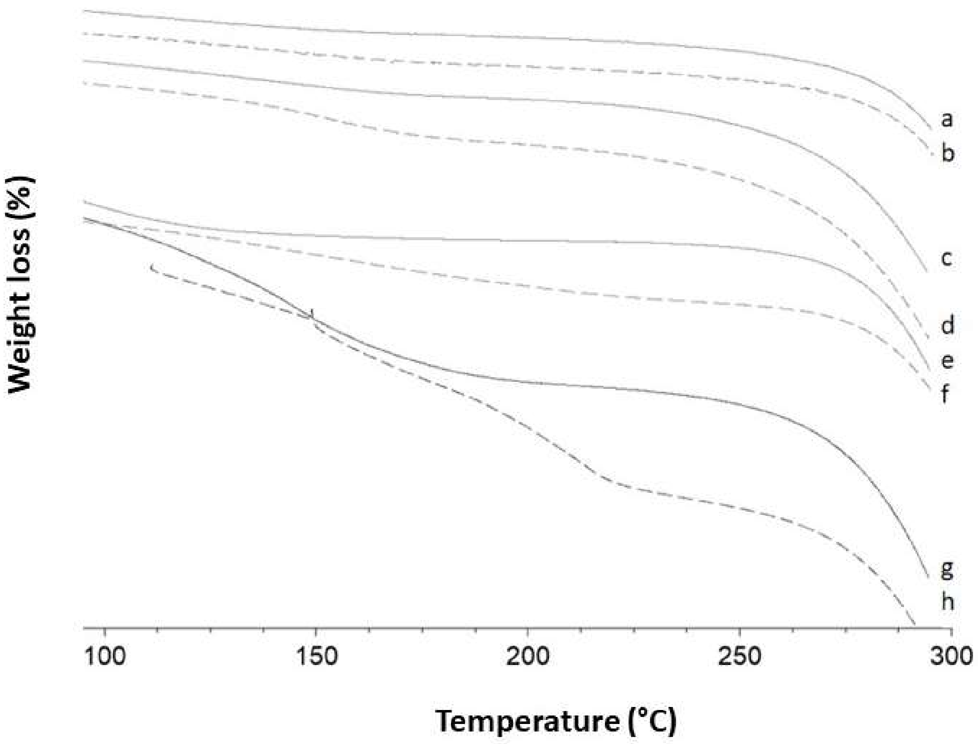

Figure S6. Thermogravimetric analysis results obtained from filaments and 3DP tablets. Key: a) PVA blank tablet; b) PVA blank filament; c) PVA NFD-loaded tablet; d) PVA NFD-loaded filament; e) HS blank tablet, f) HS blank filament; g) HS NFD-loaded tablet; h) HS NFD-loaded filament.

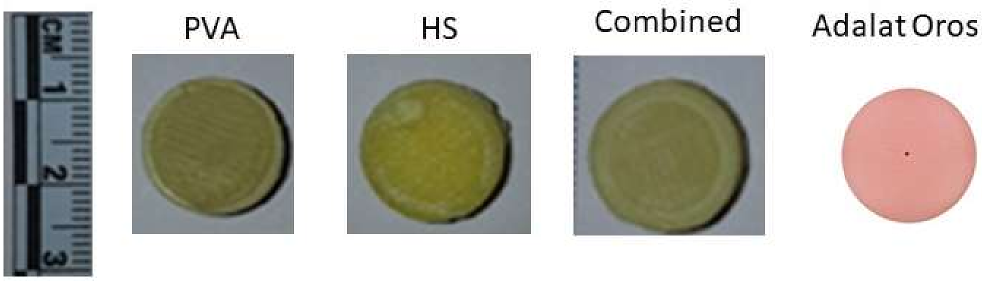

Figure S7. Size of 3DP tablets compared to Adalat Oros tablet.

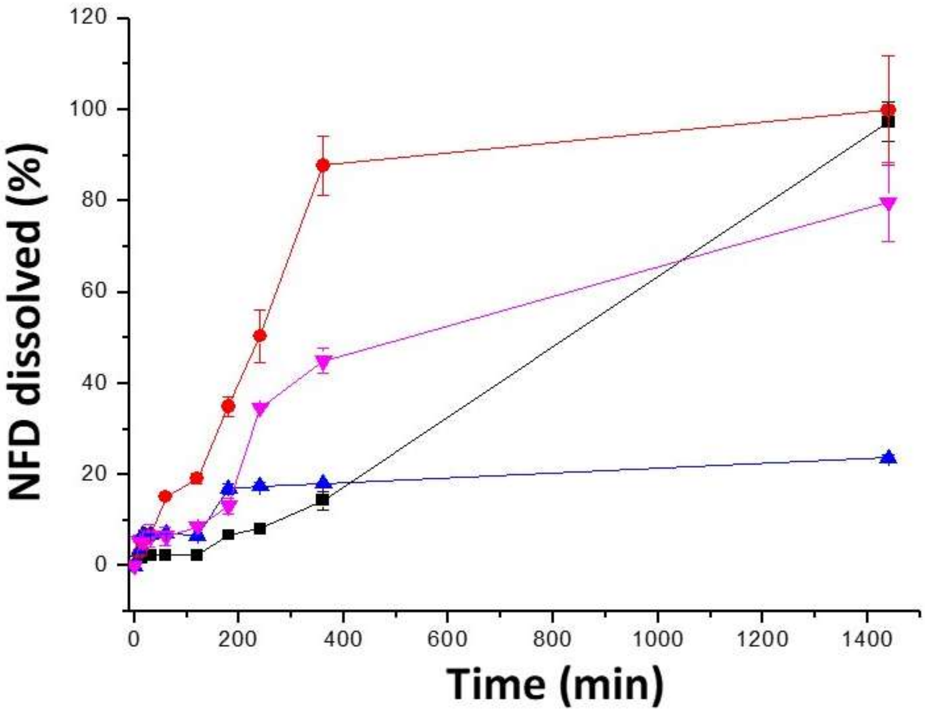

Figure S8. Dissolution profile of 3DP tablets compared to NFD commercially available formulations. Key: HS NFD-loaded tablet (red), PVA NFD-loaded tablet (blue), Combined tablet (pink), Adalat Oros (black).

Table S1. R^2 data for different kinetic dissolution models (1st 6 h).

System	Zero order	First order	Hixson-Crowell	Korsmeyer-Peppas
HS NFD-loaded tablet	0.989	0.958	0.969	0.995
PVA NFD-loaded tablet	0.899	0.906	0.904	0.933
Combined NFD-loaded tablet	0.949	0.935	0.940	0.958
Adalat Oros	0.989 (for 24 h)	0.958	0.959	0.965

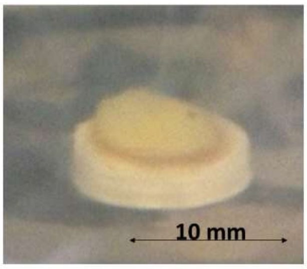

Figure 9S. Hybrid tablet during the dissolution study (after 6 h). Top surface corresponds to HS section and bottom part corresponds to PVA section.

Table S2. R^2 data for different kinetic stability models.

T (⁰C)/ RH (%)	Zero-order	First-order	Second-order	Avrami	Diffusion
80/75	0.8583	0.8624	0.8666	0.952	0.9732
80/11	0.8224	0.8304	0.8384	0.915	0.8829
70/50	0.9983	0.9969	0.9951	0.9676	0.8829
70/11	0.7215	0.7263	0.7314	0.847	0.8829
60/75	0.9931	0.9991	0.9995	0.8997	0.8829
50/50	0.9946	0.9898	0.9839	0.9372	0.8829
HS 3DP tablet	0.89803	0.90081	0.90248	0.9197	0.8979

© 2020 by the authors. Submitted for possible open access publication under the terms and conditions of the Creative Commons Attribution (CC BY) license (http://creativecommons.org/licenses/by/4.0/).
